# Predictions of Backbone Dynamics in Intrinsically Disordered Proteins Using De Novo Fragment-Based Protein Structure Predictions

**DOI:** 10.1038/s41598-017-07156-1

**Published:** 2017-08-01

**Authors:** Tomasz Kosciolek, Daniel W. A. Buchan, David T. Jones

**Affiliations:** 1 Bioinformatics Group, Department of Computer Science, University College London, Gower Street, London, WC1E 6BT United Kingdom; 2 Present Address: Department of Pediatrics, University of California San Diego, La Jolla, CA 92093 USA

## Abstract

Intrinsically disordaered proteins (IDPs) are a prevalent phenomenon with over 30% of human proteins estimated to have long disordered regions. Computational methods are widely used to study IDPs, however, nearly all treat disorder in a binary fashion, not accounting for the structural heterogeneity present in disordered regions. Here, we present a new *de novo* method, FRAGFOLD-IDP, which addresses this problem. Using 200 protein structural ensembles derived from NMR, we show that FRAGFOLD-IDP achieves superior results compared to methods which can predict related data (NMR order parameter, or crystallographic B-factor). FRAGFOLD-IDP produces very good predictions for 33.5% of cases and helps to get a better insight into the dynamics of the disordered ensembles. The results also show it is not necessary to predict the correct fold of the protein to reliably predict per-residue fluctuations. It implies that disorder is a local property and it does not depend on the fold. Our results are orthogonal to DynaMine, the only other method significantly better than the naïve prediction. We therefore combine these two using a neural network. FRAGFOLD-IDP enables better insight into backbone dynamics in IDPs and opens exciting possibilities for the design of disordered ensembles, disorder-to-order transitions, or design for protein dynamics.

## Introduction

### What are IDPs?

Intrinsically disordered proteins (IDPs) are most commonly defined as proteins which lack stable tertiary structure under physiological conditions^1–3^. It is now accepted that IDPs form a distinct group of proteins (alongside globular, transmembrane and fibrillary proteins) and are not the result of experimental procedures^1, 4–6^. Their presence *in vivo* was confirmed by NMR experiments^7, 8^.

### Abundance

Based on computational techniques, it is estimated that IDPs contribute significantly to the proteomes of different organisms^9^. In eukaryotes, around 30% of proteins have long disordered regions (i.e. more than 30 consecutive residues), while in prokaryotes the IDP abundance is lower and estimated between 1 and 7% of proteins^9, 10^. Looking at the distribution of disordered proteins between different organisms, there is a general consensus that the abundance of IDPs (especially with long disordered regions) increases in higher organisms^2, 11^. For example, in humans disordered proteins with long disordered regions are estimated at 44% of the proteome^12^. This observation is tightly linked to function of IDPs and evolution^13, 14^.

### Role of IDPs

Disordered proteins are associated with the evolutionary functional achievements of eukaryotic cells. The functional hallmark of disorder is its ability to mediate specific interaction with multiple binding partners^1, 15^. As a result, IDPs can perform molecular recognition associated with signalling and regulation, as well as binding^3, 9, 16, 17^. Because of the dynamic nature of the disordered state, IDPs provide a larger interaction surface than ordered proteins of similar size. IDPs are thus able to perform low affinity and high specificity binding^18^. From the protein interaction network perspective, IDPs are often found to be hubs of protein networks^9, 19, 20^. It has been estimated that two thirds of all signalling proteins have long disordered regions^21, 22^.

It was found that the majority of protein disease-associated mutations are found in IDPs^2, 23^. IDPs are linked to many crucial cellular functions, especially in eukaryotes. Therefore, their dysfunction or inappropriate expression can result in pathological conditions^16^. The most widespread associations between IDPs and disease are with cancers and neurodegenerative disorders^23^.

### Experimental techniques

Experimental information on IDPs come mostly from 2 techniques – X-ray crystallography and NMR spectroscopy. In X-ray crystallography, due to noncoherent X-ray scattering, disordered regions are not visible in the diffraction pattern at all, or have high crystallographic B-factor values^24^. This way X-ray crystallography accounts for the indirect evidence of disorder. NMR, unlike X-ray crystallography, is capable of producing a set of output structures (an ensemble) giving insight into the dynamics of the protein^25–28^. This wealth of dynamic information about the disordered state is not available from any other experimental technique. However, we should note that even NMR does not produce complete structural ensembles. Structures derived from NMR experiments come from an under-determined system of constraints and therefore represent one of many possible solutions^28^.

In crystallography, protein disorder is often proxied by crystallographic B-factor which is a measure of electron density spread^29^. In an experimental setting, B-factor can also indicate possible X-ray structure errors and depend on the resolution of the crystal structure, crystal contacts and on the structure refinement procedures^30^.

In NMR, order parameter (S^2^) is frequently used to characterize the level of disorder within a protein structure. The parameter itself is an experimental NMR parameter which represents how restricted is the movement of an atomic bond vector with respect to the reference frame. A value of 1 means that the movement is completely restricted (rigid) and 0 means there are no constraints on the movement of the bond (highly disordered)^31^. Experimentally, order parameters can represent movements at different time scales – from femtosecond to low millisecond^32^.

### Computational techniques

In parallel, or even at times ahead of the development of experimental techniques, there have been many attempts to study intrinsically disordered proteins computationally. The vast majority of these studies have focused on the development of disorder/order classification methods. Sequence-based disorder prediction methods treat intrinsic protein disorder in a binary fashion, residues are either classed disordered or not. Approaching disorder this way greatly simplifies the problem, as disordered regions can have many preferred conformations, functional roles and features^33^.

Apart from one-dimensional sequence-based disorder predictors, some approaches to computationally model the dynamic nature of IDPs have also been attempted. Simulations are based either on the use of all-atom molecular dynamics (MD), some form of coarse-grained molecular dynamics, or on Metropolis Monte Carlo simulations in an implicit solvent^34–38^. Although simulation techniques make it possible to study IDP systems in detail they are currently limited to small proteins or peptides (in most cases shorter than 60 residues) and require a starting experimental structure as input. Some more modern coarse grained-MD techniques (e.g. CABSflex) allow for simulations of longer proteins^39, 40^. These techniques could potentially generate complete or near-complete information on intrinsically disordered protein ensembles.

There is also a class of computational methods that give access to the information on protein dynamics indirectly, by predicting parameters which can be associated with protein backbone dynamics. Such parameters can be either crystallographic B-factors^30, 41^, or NMR order parameters^32, 42^.

### This work

In light of the exceptional role that IDPs play in organisms and experimental difficulties hindering their studies, it is important to develop computational techniques that would permit greater insight into the behaviour of this class of proteins. Here, we address the issue of the utility of protein structure prediction techniques to the *de novo* prediction of intrinsically disordered protein ensembles and protein backbone dynamics by developing FRAGFOLD-IDP. Using only sequence information we bypass the limitations of computational simulation techniques imposed by the requirement of a starting structure. And by relying on a fragment-based approach we make simulations of proteins accessible to NMR spectroscopy (up to around 200 residues) computationally tractable.

Using FRAGFOLD, a fragment-assembly protein structure prediction method, we generate raw ensembles of proteins^43–45^. Then, on those ensembles we perform clustering to extract a final ensemble. Finally, we perform sliding window superposition and analysis to obtain information on per-residue backbone fluctuations which we relate to disorder. This way, from sequence information alone, we show that it is possible to accurately predict backbone dynamics in IDPs and that our method significantly surpasses any other method producing comparable information. We also develop a machine learning-based consensus predictor, which uses FRAGFOLD-IDP and DynaMine order parameter predictions^32^ to improve protein backbone dynamics predictions even further.

## Results

**Figure 1:**
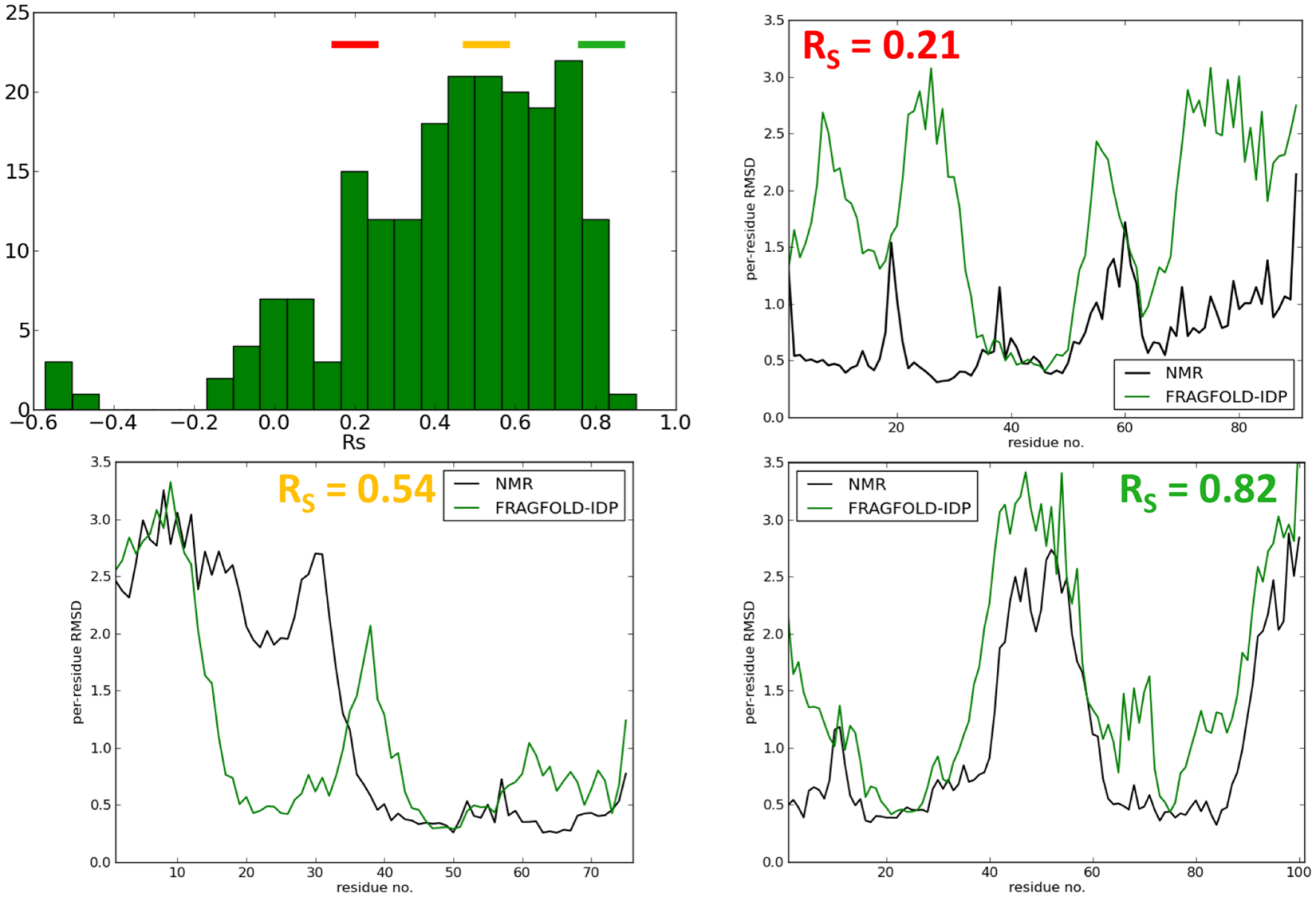
Distribution and examples of FRAGFOLD-IDP results on the 200 protein dataset. Top left panel shows the distribution of FRAGFOLD-IDP results. Sample disorder profiles of poor (top right), medium quality (bottom left) and excellent (bottom right) FRAGFOLD-IDP predictions are compared to respective NMR PDB ensembles.

**Figure 2:**
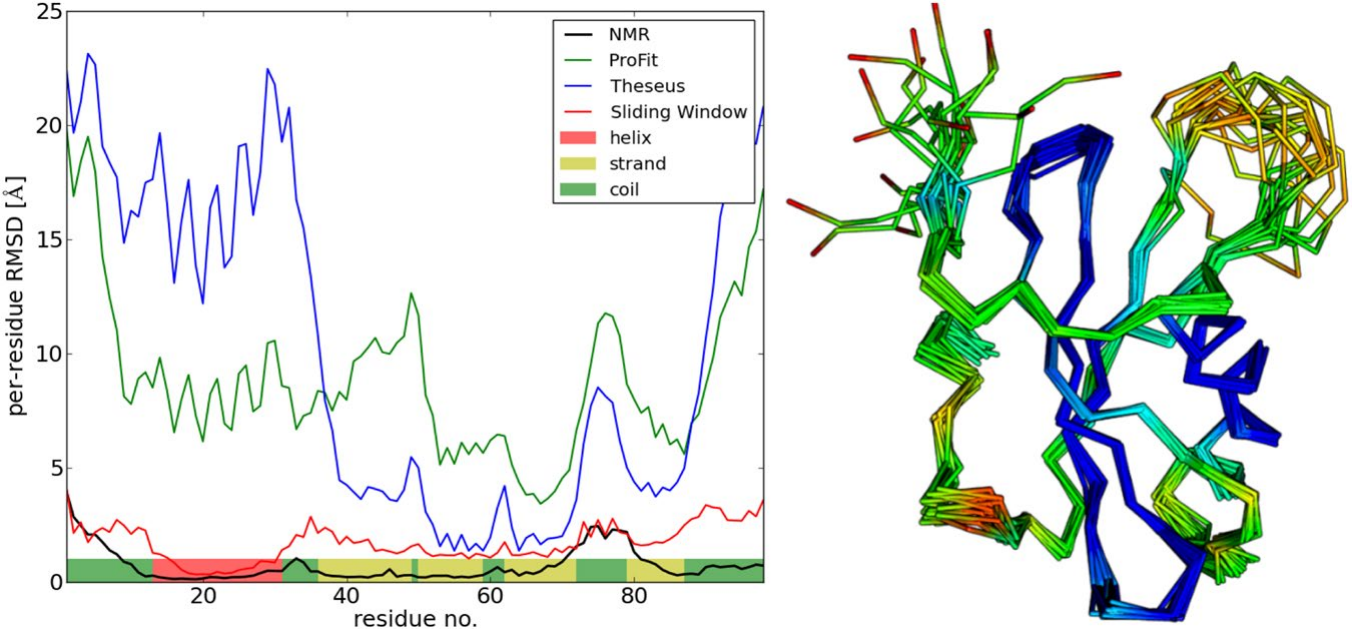
Disorder profile of stefin A (1DVD). NMR ensemble is compared with the same ensemble using alternative structural superposition methods – global superposition (ProFit and Theseus) and sliding window superposition. Predicted secondary structure elements are also highlighted. Visualisation of an NMR ensemble and FRAGFOLD predictions of 1DVD (stefin A). The original NMR ensemble deposited in the PDB was split, so that all of the conformations are overlaid onto each other. FRAGFOLD-IDP prediction is represented here by a spectrum of colours, where blue represents the most rigid predicted residues, followed by green and yellow. Orange and red represent the most disordered regions according to FRAGFOLD-IDP predictions.

In this section, we describe the results of FRAGFOLD-IDP backbone dynamics predictions on a dataset of 200 protein ensembles solved by NMR and extracted from the PDB (Fig. 1). Because we are comparing protein backbone dynamics extracted from the predicted and experimental NMR ensembles, we rely on local structural superposition achieved by the use of a sliding window. We use a sliding window of 10 residues and average per-residue RMSD values on overlapping segments. Sliding window superposition removes effects of rigid body motions and enables us to decouple structure and backbone flexibility predictions (Fig. 2). Due to this we can assess protein backbone dynamics and protein structure prediction quality separately and are not limited by the completeness of the structural ensemble generated by FRAGFOLD-IDP or coming from NMR data.

### Overview of FRAGFOLD-IDP results

FRAGFOLD-IDP uses Spearman’s rank correlation values (R_S_) as a method to score the predictions. The overall performance of FRAGFOLD-IDP on the entire dataset is presented in Fig. 1. The mean R_S_ value is 0.44 and median is 0.48. Out of 200 proteins in the dataset, 187 predictions have R_S_ > 0 (93.5%). There are 4 clear outliers, having predictions with R_S_ < −0.4 (discussed below).

R_S_ is a reliable comparative metric which works well in a qualitative setting. However, considering the predictions of protein backbone dynamics R_S_ values themselves are difficult to interpret, i.e. does R_S_ = 0.5 represent a good prediction? This difference is apparent comparing the interpretation of R_S_ values to TM-score, which has clear statistical and structural interpretation; TM-score of 0.5 and above is typically interpreted as a good prediction and the two compared proteins share the same fold^46^.

It is difficult to state the boundary between ‘good’ and ‘bad’ predictions for the problem of protein backbone dynamics predictions. In structural classification, there are terms such as class, fold or topology (and respective databases, e.g. CATH^47, 48^ and SCOP^49, 50^). In protein backbone dynamics there is still no such classification, except for descriptive identification of disordered states, such as molten globule, entropic chain, etc.^33^.

Nevertheless, from the visual analysis of the results and from previous studies attempting to predict 1-S^2^ (NMR order parameter) from NMR ensembles, some intuition can be derived. Zhang & Bruschweiler derive an analytical expression to calculate the order parameter from NMR and X-ray structures^51^. Their method achieves a mean R_S_ value of 0.61 comparing S^2^ values calculated from X-ray structures against experimental S^2^ values for 5 proteins and a mean of 0.67 for comparisons with NMR structures on the same set. However the test set is small, the results suggest that R_S_ values of above 0.6 indicate very good predictions.

Another way to estimate R_S_ values typical of ‘good’ predictions is to compare them to some other prediction methods. One of such methods is CABSflex^39, 40, 52^. It is a coarse-grained method that attempts to predict protein backbone dynamics from a single structure. It was shown to perform very well in comparison with both NMR and MD results^39^. Unlike FRAGFOLD-IDP, CABSflex has a significant advantage by starting from a known structure. Therefore, it can be assumed that CABSflex predictions should constitute what can be assumed excellent FRAGFOLD-IDP predictions. Since the entire dataset used in this study has corresponding experimental structures, for each case in the benchmark set a single structure (MODEL 1) was extracted from the PDB file and submitted to the CABSflex server (http://biocomp.chem.uw.edu.pl/CABSflex/)^52^. After obtaining CABSflex per-residue RMSD predictions, the results were again evaluated using R_S_. Mean R_S_ achieved this way on the benchmark set is 0.66 (median 0.70). The results are close to the ones reported in the CABSflex paper, comparing CABSflex simulations to NMR per-residue fluctuations using RMSF, instead of RMSD^40^. The paper reports R_S_ values = 0.72 (±0.15). Again, this confirms that R_S_ values of around 0.6 could be considered typical of very good predictions and 0.7 and above, excellent predictions.

Equipped with intuition as to how to interpret the R_S_ values, we can discuss FRAGFOLD-IDP performance in more detail (Fig. 1). There are 67 very good predictions with R_S_ ≥ 0.6 (33.5%). They include 35 excellent predictions with R_S_ ≥ 0.7 (17.5%). Examples of some poor, good and excellent predictions are presented in Fig. 1 and discussed in Supplementary Information (Supplementary Text S1). A sample FRAGFOLD-IDP prediction along with a 3D visualisation is shown in Fig. 2.

An interesting aspect of the initial results are also the 4 outliers in the distribution (Fig. 1; R_S_ < −0.4). Two of the cases among the outliers are clearly related to the ensemble extraction method – 1XN7 and 2K02 (Supplementary Text S2). The final FRAGFOLD-IDP ensemble results are low, but among FRAGFOLD-generated models (raw ensembles) there are some with excellent R_S_ values. The remaining cases – 1G6M and 1K0T – are more challenging. From the NMR PDB ensemble of 1G6M it can be inferred there are 4 disulphide bridges that constrain the structure making it more ordered (Supplementary Text S2). Those bridges are the likely cause of poor predictions of the backbone dynamics achieved by FRAGFOLD-IDP. The second protein, 1K0T, also exhibits some structural difficulties. Although 1K0T passed all of the dataset criteria (see Materials & Methods), it has two inorganic clusters (Fe_4_S_4_) covalently bound to the protein (Supplementary Text S2). Such modification is likely to alter backbone dynamics of the protein. It can also be confirmed by the fact that other backbone dynamics predictors evaluated fail to considerably improve (e.g. DynaMine R_S_ = 0.23) over FRAGFOLD-IDP predictions.

### Impact of structure prediction quality on backbone dynamics predictions

**Figure 3:**
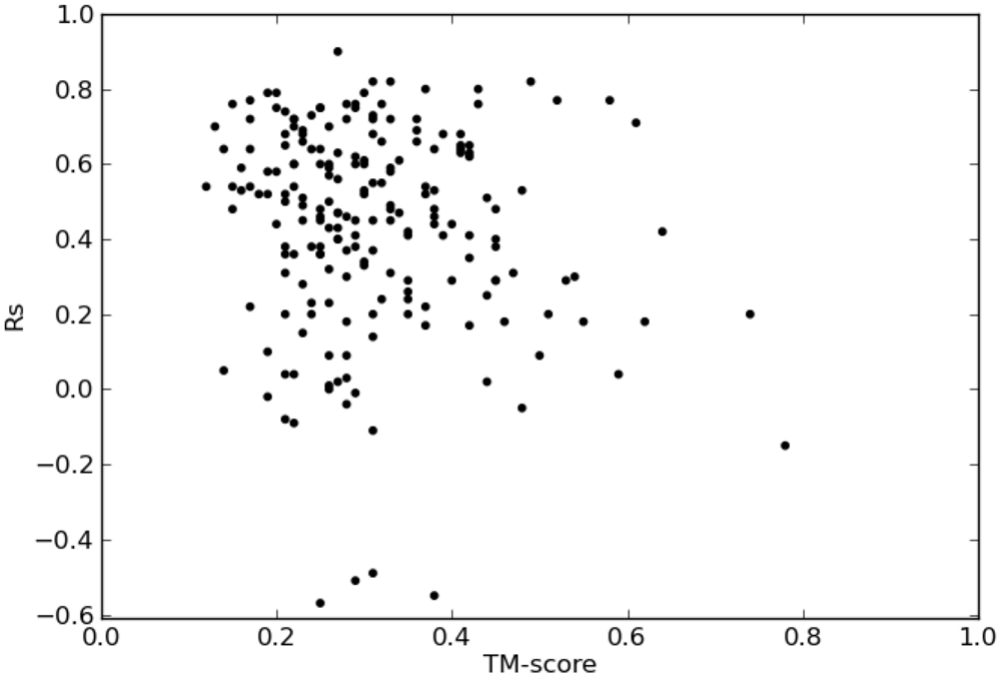
Impact of structure prediction quality (TM-score) on backbone dynamics predictions (R_S_).

In FRAGFOLD-IDP we introduce the idea of separating protein backbone dynamics from protein structure predictions. Having established FRAGFOLD-IDP and taking advantage of this separation, we can now compare whether the quality of backbone dynamics predictions depend on the quality of structure predictions. To do this, the TM-score for each protein in the dataset was calculated as described in Materials & Methods section. Each protein is now characterized by 2 values – the TM-score representing the structure prediction quality between the 2 ensembles (FRAGFOLD-IDP and NMR PDB) and R_S_ reflecting the quality of backbone dynamics predictions (Fig. 3). A protein with both R_S_ and TM-score values close to 1, would represent an ensemble structurally and dynamically similar to its NMR counterpart, but would not necessarily mean that any of those ensembles is complete.

The immediate conclusion is that it is not necessary to find the correct fold of the protein to predict its backbone dynamics accurately. Posing an alternative hypothesis is more challenging – does high structure prediction quality (high TM-score) hinder the predictions of backbone dynamics (low R_S_ values)? The analysed dataset is under-represented in well-folded (TM-score ≥0.5) structures. Only 8% of the dataset (16 structures) has TM-score ≥0.5. In comparison, previous work which concentrated on predicting the structures of globular proteins found that FRAGFOLD is able to correctly predict around 14–25% of cases, depending on the final model selection criteria^45^. Because of the under-representation of high quality structure predictions, it is difficult to draw robust conclusions based on this dataset.

The overall relationship between how a correct structural model and disorder profile remains unclear (Fig. 3). The data shows almost no correlation between the TM-score and R_S_. Similar behaviour is observed regardless ensemble extraction method, e.g. analysing the data for the best cluster (instead of selecting the largest cluster).

To investigate this further, we also tested an alternative approach. TM-score is a continuous measure, so a TM-score of 0.2 indicates a poorer model than observing a TM-score of 0.3. Nevertheless two such models would be unsatisfactory predictions. Following this rationale, TM-score values were binned and the correlations recalculated. The bin boundaries were established based on the findings of Xu & Zhang^46^. The first bin contains TM-score values from 0 to 0.2, corresponding to random non-homologous structures. The second bin includes ensembles with TM-score between 0.2 and 0.4 TM-score – values where the posterior probability of 2 structures belonging to the same CATH or SCOP class is close to zero. The third bin contains TM-scores between 0.4 and 0.6 – the “phase transition” region, where the probability of the two protein belong to the same fold increases drastically and reaches around 90%. The last bin includes ensembles with TM-score above 0.6 and contains cases where the posterior probability of the two proteins/ensembles belonging to the same fold is >90%. The bin boundaries were also validated to maximize the Pearson’s r correlation value between TM-score and R_S_.

Even applying this binning protocol does not provide reliable answers. The 162 targets which belong to bin 1 or 2 have FRAGFOLD-IDP ensembles that are unlikely to belong to the same CATH or SCOP class as their NMR PDB counterparts. Mean R_S_ values in bins 1 and 2 are on average higher than the ones in bins 3 and 4. There are 33 proteins in bin 3 and only 5 proteins in bin 4, with a total of 14 All-alpha proteins, 11 All-beta, 11 Alpha/beta and 2 No Class proteins. Comparing the enrichment of protein populations in the two top bins (3 and 4), the bins are most enriched in All-beta (1.93) and All-alpha (1.27) proteins. Alpha/beta proteins are proportionally represented and No Class proteins have reduced representation (0.23) in the top two bins. The All-alpha and low secondary structures classes generally show higher than an average R_S_ values, whereas All-beta and No Class proteins perform below average in terms of R_S_. Hence, one of the possibilities of why bins 3 and 4 show lower R_S_ predictions, is that they are highly enriched in all-beta proteins. However, bins 3 and 4 (high TM-score ensembles) are under-represented in the set (38 protein in total) and some of the best scoring targets in terms of TM-score are outliers in terms of their backbone dynamics predictions (e.g. 1G6M discussed previously). Hence, the decrease of R_S_ values in bin 4 is unlikely to be a significant effect.

### Comparison of FRAGFOLD-IDP with other computational techniques

For every newly developed computational method, it is desirable to make comparisons to other state-of-the-art approaches to determine how the new method performs and identify its strengths and weaknesses among the available computational techniques. As a naïve method we used PSIPRED secondary structure predictions and assigned arbitrary per-residue RMSD values to helix, strand and coil predictions. It can tell us whether a computational method does any better than to indirectly infer secondary structure and assume that all loops are disordered. The comparison to other state-of-the-art methods is difficult, since the only other approach to predict protein backbone dynamics from sequence is DynaMine. Notably, DynaMine does not predict protein backbone dynamics directly, but approximates it using NMR order parameters. As a machine learning-based method, it was trained on order parameter values derived from chemical shifts, so that predicted values represent a mix of different timescales^32^.

To increase the variety of computational methods, we also included some other approaches that provide related information, i.e. crystallographic B-factor predictors and disorder/order predictors which were also shown to contain information related to protein backbone dynamics^53^.

The comparison between FRAGFOLD-IDP, DynaMine^32, 42^, PROFbval^41^, DISOPRED3^54^ and IUpred^55, 56^ was carried out on the 200 protein dataset used throughout this paper. DISOPRED3 and IUpred were selected to represent the disorder predictors, as they reflect the two main approaches to disorder/order classification – machine learning-based (DISOPRED3) and statistical energy-based (IUpred).

**Figure 4:**
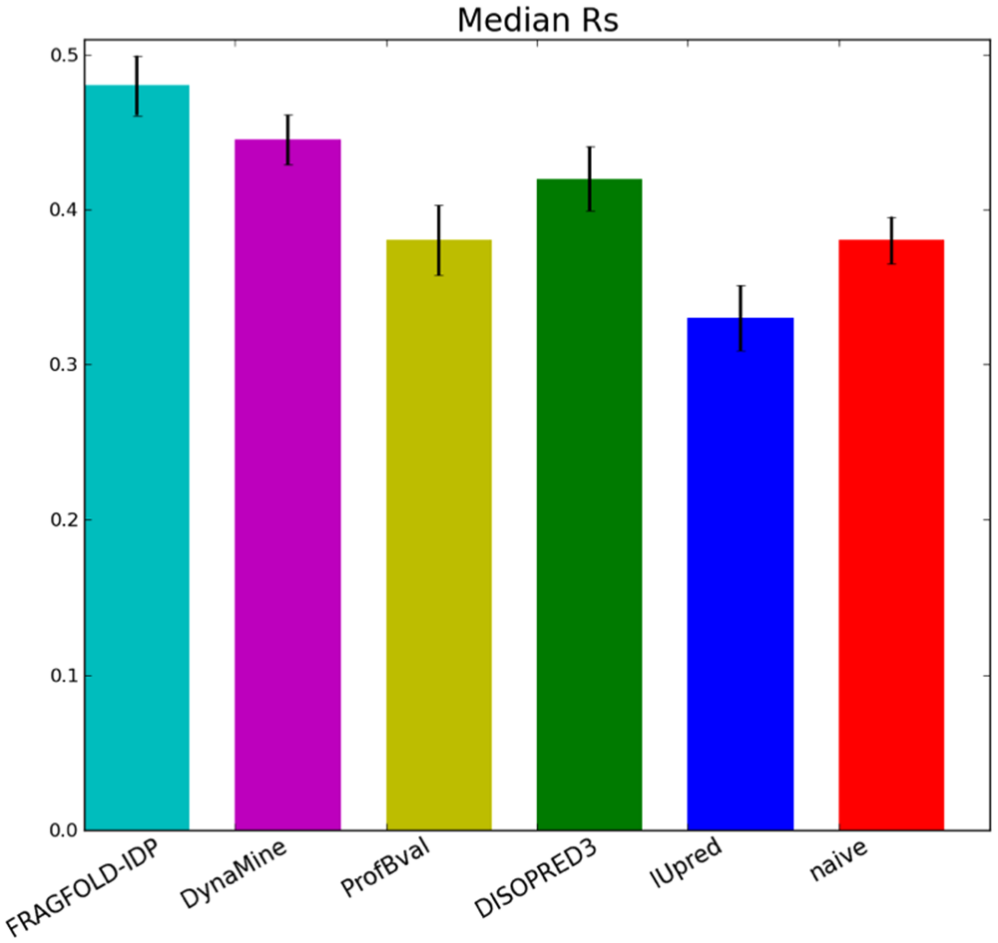
Median R_S_ values between FRAGFOLD-IDP and other computational techniques.

The results of the predictions are presented in Fig. 4. The chart uses median R_S_ values for comparison, because it is a more robust metric than the average, especially in the presence of outliers. Overall, FRAGFOLD-IDP and DynaMine clearly perform best and significantly better than the naïve method (Wilcoxon signed-rank test p-value = 0.004). PROFbval predictions and IUpred achieve performance on par with the naïve approach. DISOPRED3 achieves higher median R_S_ than the naïve, but the result is not statistically significant (p-value = 0.73).

Because the only computational techniques that achieve results significantly higher than the naïve approach are FRAGFOLD-IDP and DynaMine, let us compare the results of those methods in more detail.

**Figure 5:**
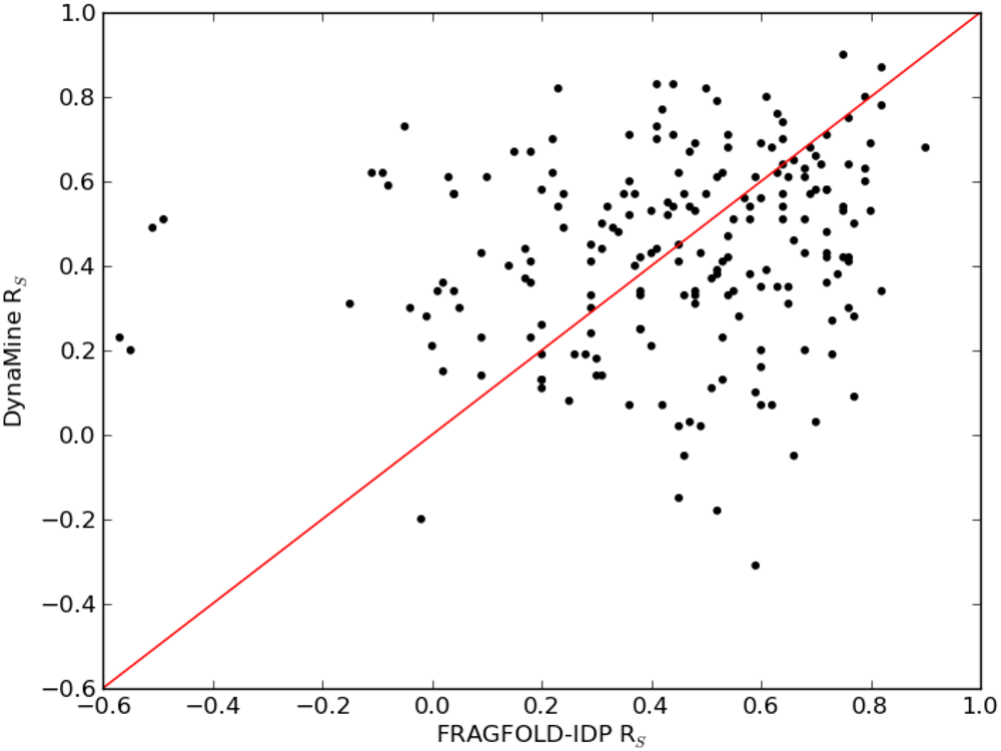
Per target comparison of FRAGFOLD-IDP and DynaMine results.

The results of FRAGFOLD-IDP and DynaMine are comparable in terms of their overall performance. Median FRAGFOLD-IDP R_S_ is 0.48 (mean R_S_ = 0.44), whereas median DynaMine R_S_ is 0.45 (mean R_S_ = 0.44). Analysing the results on a per case basis, FRAGFOLD-IDP achieves higher R_S_ for 109 out of 200 cases (Fig. 5). But more interestingly, the results of the two methods are very weakly correlated (r = 0.17, p-value = 0.013), even when FRAGFOLD-IDP outliers are removed (p-value goes up to 0.015).

The lack of correlation (or very weak correlation) between the FRAGFOLD-IDP and DynaMine results suggests that the methods in a practical setting could complement one another. The results also suggest that for the most part poor results achieved by FRAGFOLD-IDP are not a cause of some experimental bias (apart from the outliers discussed previously), but rather that FRAGFOLD-IDP is unable to cope with them effectively.

We attempted to find some indicators as to what determines if FRAGFOLD-IDP or DynaMine perform well. We were not able to find any clear signals, such as physicochemical properties, disorder or secondary structure content, or the fold of the protein which would indicate when to use one method over the other. FRAGFOLD-IDP has below average performance for mostly beta CATH class proteins and for alpha/beta and few secondary structures classes, it seems to decrease its performance with the increase of disorder content (Supplementary Fig. S1). This however lacks robustness, as the dataset is under-represented in proteins with disorder content above 60%. Therefore, to leverage the orthogonality of FRAGFOLD-IDP and DynaMine we constructed a consensus protein backbone dynamics predictor. The predictor uses the outputs of these two methods as inputs to further improve the backbone dynamics predictions and take advantage of the strengths of both of FRAGFOLD-IDP and DynaMine.

### Consensus protein backbone dynamics predictor

**Figure 6:**
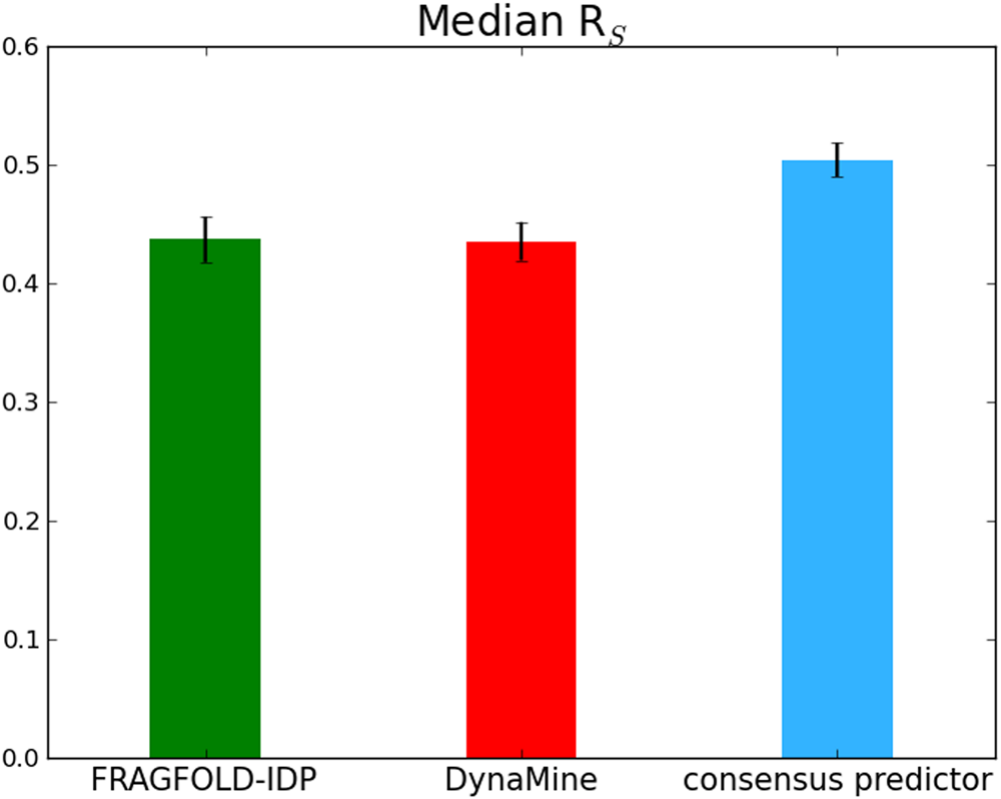
Comparison of FRAGFOLD-IDP, DynaMine and consensus predictor median R_S_ values.

The consensus backbone dynamics predictor is based on a neural network architecture. The results of the consensus predictor come from cross-validation (described in Materials & Methods section). Comparing median R_S_ values obtained on the 200 NMR PDB dataset, the consensus predictor quite clearly improves over both FRAGFOLD-IDP and DynaMine (Fig. 6).

FRAGFOLD-IDP achieves higher median R_S_ (0.48) than DynaMine (R_S_ = 0.44), but the differences are not significant (Wilcoxon signed-rank test p-value = 0.63; Fig. 6). In comparison, the consensus predictor achieves median R_S_ = 0.54 and those results are significantly better than both DynaMine and FRAGFOLD-IDP (Wilcoxon signed-rank test p-value < 0.001 for both methods).

Interestingly, the results of both input methods (FRAGFOLD-IDP and DynaMine) were not correlated (r = 0.17), but the results of the consensus predictor are correlated with both FRAGFOLD-IDP (r = 0.57) and DynaMine (r = 0.65). This shows that the consensus predictor was able to extract top results from both approaches, still significantly improving over any of them.

Also, looking at the number of ‘good’ (R_S_ ≥ 0.6) and ‘excellent’ (R_S_ ≥ 0.7) predictions, the consensus predictor performs well (Table 1). It significantly improves over both input methods in terms of the number of very good predictions (R_S_ ≥ 0.6), achieving 77 such results. But in terms of excellent predictions (R_S_ ≥ 0.7) it performs slightly worse than FRAGFOLD-IDP alone (30 in the consensus predictor and 35 in FRAGFOLD-IDP). The likely cause of the drop in the number of excellent predictions is the relatively large discrepancy in the number of FRAGFOLD-IDP and DynaMine predictions in this class (Table 1). Although the consensus predictor improves over both input methods, it is still constrained by the results provided by FRAGFOLD-IDP and DynaMine as inputs.

**Table 1:** Good and excellent predictions produced by the algorithms.

R_S_	FRAGFOLD-IDP	DynaMine	consensus predictor
<0	12	6	2
≥0.6	67	54	77
≥0.7	35	22	30

The predictions produced by the consensus predictor are also more conservative and there are only 2 cases with R_S_ below 0 (Table 1). Notably, the consensus predictor is able to remove all of the outliers produced by FRAGFOLD-IDP.

**Figure 7:**
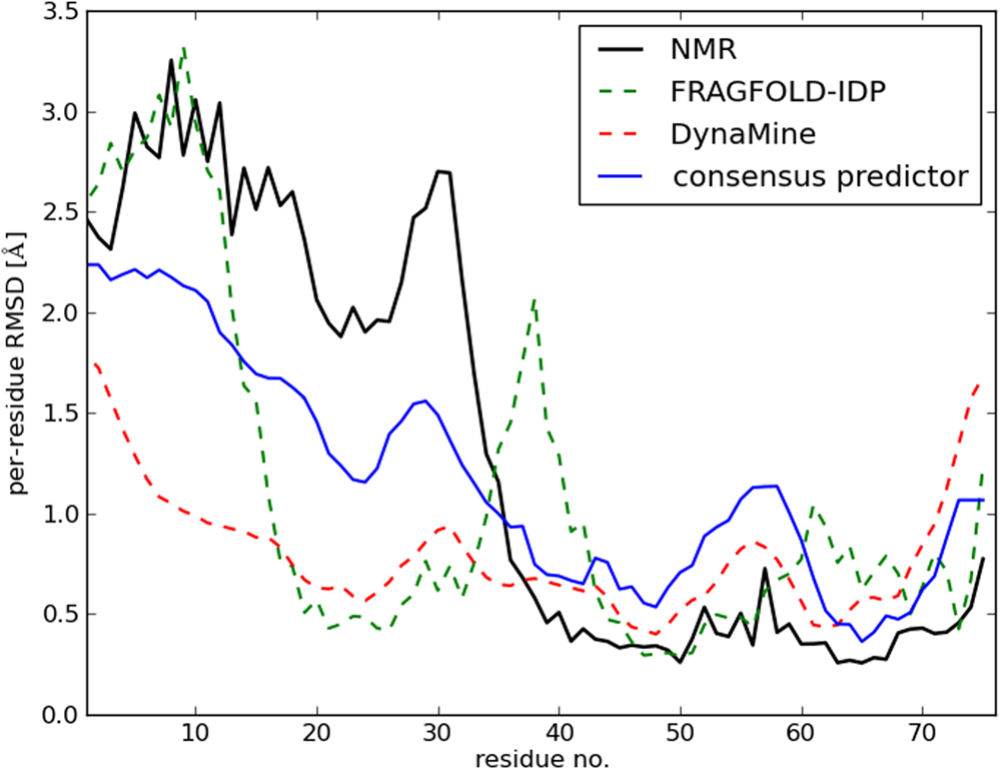
Example of an excellent consensus predictor result – 1P94. DynaMine (R_S_ = 0.71) and FRAGFOLD-IDP (R_S_ = 0.54) produce good predictions for this target. Consensus predictor performs remarkably well (R_S_ = 0.87).

A good example of a target where the consensus predictor works well, improving over both input methods and taking advantage of the strengths of both approaches is 1P94 (Fig. 7). The consensus predictor achieves an excellent result on this target (R_S_ = 0.87). FRAGFOLD-IDP (R_S_ = 0.54) correctly identifies part of the highly disordered N-terminal region (up to residue 15) and the ordered part of the protein between residues 48 and 76. DynaMine performs better (R_S_ = 0.71), but also fails to identify the behaviour of the protein in the highly disordered regions between residues 1 and 35. Also, the region between resides 25 and 35 is predicted to exhibit similar behaviour as the region between residues 50 and 60. Similarly, residues 1–5 and 70–76 show near identical behaviour, while the NMR ensemble shows that the N-terminus is highly disordered, and the C-terminus is ordered. The concerns about the behaviour of DynaMine partly stem from the fact that DynaMine predicts order parameters, not per-residue RMSD. The results shown in Fig. 7 are scaled results of 1-S^2^ (DynaMine predictions).

The consensus predictor performs remarkably well on this target. Although the per-residue RMSD values do not match exactly (they were back-calculated from 0 to 1 values using an inverse logistic function), all of the features of the NMR ensemble are captured (Fig. 7). The long disordered region between residues 1 and 35 is reproduced well – the consensus predictor values are highest in this region (i.e. higher than between residues 50 and 60, or in the C-terminus region). This includes the trough between residues 20 and 30 and the per-residue RMSD values which are higher between residues 1 and 10 than between residues 25 and 30. Also, the short region of elevated per-residue RMSD between residues 50 and 60 is reproduced accurately. Contrasting the predictions of the consensus predictor with those of FRAGFOLD-IDP and DynaMine it is clear that the predictor goes beyond simply combining the results of the input methods. For example, let us consider the region around residue 20, including the trough around residue 25. Both FRAGFOLD-IDP and DynaMine predict that the region around residue 25 has relatively low per-residue RMSD. But considering its immediate environment, both input methods over-predict its breadth, while the consensus predictor is able to correctly find the behaviour of the disorder profile between residues 20 and 25. Also, according to both input methods, the trough at residue 25 shows per-residue RMSD values lower than the region between residues 50 and 60. The consensus predictor is also able to rectify this mistake and correctly assign per-residue RMSD values as higher than between residues 50 and 60 (and above 70, where DynaMine and FRAGFOLD-IDP also fail).

## Discussion

In this work, we confirm that the *de novo* predictions of protein backbone dynamics are possible. Hence, this property is encoded in the protein sequence, similarly to disorder as a state.

Protein intrinsic disorder is a state related to protein function^1, 15, 16^. It is not a binary property and not all conformational states are permitted in disordered ensembles. Disease-associated mutations need not cause disorder-to-order transitions^57, 58^. The majority of disease-associated mutations can be classified as disorder-to-disorder transitions, likely impacting the ability of the protein to interact with its binding partners, or changing the properties of the disordered ensemble. Therefore, going beyond the binary disorder-order classification is indispensable to be able to grasp the impact of those changes. Accurate predictions of protein backbone dynamics may open up the possibility to study the changes of the disordered state in response to external factors i.e. to perform disorder design and, in future, other biomedical applications such as the design of small molecules to alter the disordered state^59, 60^.

In more general terms, protein backbone dynamics predictions could be related to functional information in proteins. Such predictions could either serve as a source of information for protein function prediction methods, or be used to guide experiments aimed at investigating the structure and function of those proteins deemed likely to be disordered.

We showed that in FRAGFOLD-IDP it is not necessary to find the correct fold of the protein in order to be able to predict its backbone dynamics accurately. From a computational perspective, it can be interpreted that in FRAGFOLD-IDP, during the folding process (i.e. FRAGFOLD simulations), only local conformations play an important role in the outcome of the calculations. Looking at the problem biologically, the results suggest that disordered regions form early in the folding process and the final conformation reached during folding does not substantially impact the disordered region. Alternatively, it could speculated that disorder is an intrinsic local property of the sequence.

During DynaMine optimisation it was found that using a wider sequence window as an input for the predictor increases the correlation between DynaMine predictions and reference experimental data^32^. However, the improvements are significant up to a window size of 23 (11 residues on either side of the residue of interest). As the authors themselves point out, the residues in the immediate neighbourhood have the greatest impact on the backbone dynamics. Hence, the conclusions from DynaMine also confirm the notion of the locality of intrinsic protein disorder.

This finding not only serves as an important observation in terms of expanding our understanding of the protein folding process, but it could also help the possible future development of FRAGFOLD-IDP directly. The computational time needed for simulating long sequences is substantial and increases exponentially with the length of the sequence. Since it is not necessary to find a correct fold for the sequence, overlapping sequence fragments could be simulated independently and then the disorder profiles assembled from the fragments. This could make long sequences accessible to FRAGFOLD-IDP simulations and could also reduce the computational time needed to obtain results.

In this work we show that FRAGFOLD-IDP and the consensus predictor we developed are the only methods that provide high quality protein backbone dynamics predictions for intrinsically disordered proteins. The predictions of protein backbone dynamics add another dimension to our knowledge about proteins. As with any computational approach, it is limited by the availability and reliability of the experimental data at hand. Disorder is a prevalent phenomenon that is notoriously difficult to grasp experimentally^1^. Several experimental techniques which are used to study ordered proteins largely fail when it comes to intrinsically disordered proteins (e.g. X-ray and EM). Further complicating the study is the observation that disorder is a metastable state susceptible to the changes in the environment. The behaviour of IDPs is often controlled by post-translational modifications, such as phosphorylation^61^. They can cause disorder-to-order or order-to-disorder transitions and alter binding affinities. Molecular crowding of the cellular environment can also impact the conformational ensembles of intrinsically disordered proteins, as it was proven by both NMR experiments^62^ and MD simulations^63^. Hence, even in cases where intrinsically disordered proteins were treated in a binary disorder/order fashion, it was shown that the classification of residues can change upon environmental variations in the experimental conditions^64^.

An exciting future application of FRAGFOLD-IDP that we envisage is its use in disorder design, i.e. conformational transitions in IDPs (either disorder-to-order, or order-to-disorder) upon amino acid substitution. Disorder/order classification methods generally perform poorly on the disorder design task^65^. There is only some anecdotal evidence of success coming from single predictions on individual proteins using single sequence-based predictors that such design is possible^57, 66, 67^. It is not surprising, as most of the disorder predictors use sequence profiles to perform the predictions and point mutations do not impact the sequence profile significantly. FRAGFOLD-IDP does not explicitly rely on profile information while predicting the dynamics. Still, one of possible obstacles in doing so is the relative paucity of data. The largest known study to date used only 31 proteins (101 mutations) with only 3 cases of disorder-to-order transitions^65^. A problem related to protein design, which could be more computationally accessible is the design for protein dynamics. It is hypothesized that proteins are not only subject to selective pressure based on their structural properties, but also their local dynamic properties. An example of this is the DHFR protein family^68^. *E. Coli* DFHR and human DHFR share considerable structural similarity, but because of different dynamic properties it was shown that human DHFR cannot substitute its homolog in bacterial cells. The design for dynamics could therefore be an interesting intermediate step towards disorder design. Here, more substantial sequence changes are observed which trigger some changes in protein disorder (dynamics) profiles.

## Materials and Methods

### Dataset

Because this work moves away from the binary disorder/order classification, we use NMR PDB ensembles, instead of relying on the classical DisProt dataset^69^, or missing electron densities from X-ray data (e.g. as in DISOPRED2^9^). The dataset was extracted from mobiDB database version 1.2^70^. The database was queried to extract only the proteins: (1) solved by NMR; (2) that have at least 95% coverage of PDB sequence with UniProt; (3) between 50 and 150 amino acids long; (4) that have no other molecules in the PDB file, as indicated by COMPND PDB keyword; (5) that have at least 5 consecutive disordered residues, as indicated by MOBI method^71^. Applying these criteria resulted in a dataset of 200 proteins. The average protein length is 105 residues and the average disorder content is 33.7%. There are 28 proteins (14%) with at least 50% of disorder content and 3 fully disordered proteins. The disorder distribution is close to what is predicted for the human proteome^11^.

### FRAGFOLD protein structure predictions

FRAGFOLD is a state-of-the-art *de novo* fragment-assembly method for protein structure prediction^43, 44, 72, 73^. It was shown to be effective in the *de novo* structure prediction of globular proteins, as it was confirmed in several CASP experiments^43, 73, 74^.

FRAGFOLD assembles folds from a mixture of supersecondary structural fragments and short fixed length fragments taken from a library of highly resolved protein structures using a simulated annealing approach. To guide the selection of fragments, FRAGFOLD relies on a multiple-sequence alignment (MSA) and secondary structure predictions provided as input. Secondary structures were generated using PSIPRED^75^ and MSAs using HHblits^76^.

FRAGFOLD uses a knowledge-based force field composed of pair-wise potentials determined by inverse Boltzmann equation, solvation potential, hydrogen bonding, structure compactness and steric terms^43^. All simulations were run using all-atom representations, Replica Exchange Monte Carlo to search for low energy conformations and relative weighting of the energy function terms determined by considering the standard deviations of each term across an ensemble random chain conformations for the target, as described by Jones, *et al*.^44^. The default parameters were used and the number of annealing steps was set to 10,000,000. 200 models per protein were generated to ensure a reasonable sampling of conformational space.

### FRAGFOLD-IDP workflow

The FRAGFOLD-IDP workflow involves 3 steps: generating raw ensembles, final ensemble extraction and assessment of the results.

For each protein sequence, an MSA and a secondary structure prediction are generated. They serve as an input to FRAGFOLD. The set of output FRAGFOLD structures constitutes the raw ensemble. This raw ensemble needs to be processed to extract what would be a final ensemble and the result of the method. We perform ensemble extraction using PFClust^77, 78^. RMSD is used as the distance metric and the largest cluster is selected as the final FRAGFOLD-IDP ensemble. The final ensemble is compared to its experimental counterpart by generating a per-residue RMSD profile using a sliding window superposition with a window size of 10 residues. In contrast with other approaches (global superposition, e.g. least squares – ProFit (http://www.bioinf.org.uk/software/profit/) or maximum likelihood – Theseus^79, 80^), sliding window superposition removes rigid body motions and enables an independent assessment of structure and backbone flexibility predictions (Fig. 2). Disorder profiles are then assessed on the basis of Spearman’s rank correlation coefficient (R_S_).

FRAGFOLD-IDP software including the consensus predictor, and example data is available for download from https://github.com/psipred/fragfold_idp.

### Naïve disorder assignment

Since Spearman’s rank correlation (R_S_) is used as the disorder match metric used for assessment in this work, the order of fluctuations along the backbone is important, but not their absolute values. As a baseline method we used predicted secondary structures, relying on the assumption that only the protein sequence is known. The secondary structure predictions were carried out using PSIPRED^75^. Having tested a set of alternative hypotheses, we have found that assuming the largest degree of flexibility within predicted loop regions (C), medium-level of flexibility in sheets (E) and low flexibility within helices (H) provided highest cumulative and mean R_S_ values calculated on the dataset.

### TM-score structure prediction quality calculations

TM-score is a robust, length-independent protein structural similarity metric^81^. It is routinely used in many structure prediction problems^45^. The higher the TM-score is (on the scale of 0 to 1), the closer is the predicted structure to its experimentally-solved equivalent. Generally, it is assumed that structures with TM-score 0.5 or higher have a correct fold and can be considered successful predictions^46^.

For the current problem, because ensemble versus ensemble comparisons are performed, TM-scores need to be calculated differently and accumulated accordingly. For each structure in the predicted ensemble, TM-score was calculated against each of the NMR models included in the PDB file. For each structure in the final FRAGFOLD-IDP ensemble, the highest TM-score was selected. As all of the structures in the predicted ensemble had their TM-scores calculated, the mean TM-score was then computed.

This averaging procedure was performed to account for the fact that FRAGFOLD ensembles don’t necessarily include all of the conformational states included in the NMR ensemble. Also, the NMR ensemble may not include every naturally occurring conformation, as it itself rather represents one of the sets of conformations that fit the experimental data.

### Third-party methods

#### DynaMine

The predictions were run using the August 2014 version of DynaMine client script from the authors’ website (http://dynamine.ibsquare.be).

#### PROFbval

The method was downloaded from the Debian repository and used with default parameters.

#### DISOPRED3

Version 3.16 with default parameters was used.

#### IUPred

Version 1.0 with default parameters was used.

### Consensus predictor architecture

The consensus predictor is a classical feed-forward neural network with a bias unit in input and hidden layers. A sliding window on input features is used. There are 27 window features and 3 global features. Window features: FRAGFOLD-IDP result (1), DynaMine result (1), amino acid composition frequency (21), PSIPRED secondary structure probabilities (3), missing residue (3). Global features: log sequence length (1), number of residues from the termini (2).

Using a sliding window of 9 residues, there are 246 input features per residue. One hidden layer and a single output unit was used. A set of alternative numbers of hidden units were tested: between 10 and 200 hidden units.

### Consensus predictor training

The network was constructed and trained using the PyBrain Python library. Because of the relatively small dataset size (200 proteins), the method was cross-validated, instead of creating separate training and test sets. To avoid overtraining, the cross-validation was performed on the basis of CATH classification^47, 48^, separating the proteins at the fold level. It is a rigorous criterion that ensures the proteins share no significant structural similarity, regardless of their disorder content. Some proteins in the dataset were not classified in CATH (45 cases). Those examples, for the purpose of cross-validation, were assigned to the CATH fold with which they share the highest similarity (lowest RMSD). All singletons were clustered together to form a separate class for cross-validation. The procedure resulted in 33 sets.

The training was performed on each class to minimize the mean squared error value. It was carried out until convergence with 20% of input data used for validation.

The network showed no substantial window size dependency on the quality of predictions, regardless of the number of hidden units, or scoring (Supplementary Fig. S2). Hence, the behaviour of the consensus predictor is substantially different to that of DynaMine^32^, where the authors observed a significant dependency of the predictions on the window size used (up to around 23-residue window). This behaviour of the consensus predictor is likely caused by the fact that the most important sources of information, i.e. DynaMine and FRAGFOLD-IDP results, were already extracted using a sliding window approach. Here, only a small window is necessary to account for the immediate sequence and physicochemical environment.

The network was trained on a number of different hidden units, ranging from 10 to 200 (Supplementary Fig. S3). As in the case of optimising the window size, there is no substantial dependency of the results on the number of hidden units. Hence, the criterion by which the final network was selected was to minimize the probability of over-training the network and 10 hidden units were selected as the optimal network size.

### Data availability

The datasets generated during and/or analysed during the current study are available from the FRAGFOLD-IDP repository, https://github.com/psipred/fragfold_idp.

## Electronic supplementary material

Supplementary Information
