# Supplementary material for: Predictions of Backbone Dynamics in Intrinsically Disordered Proteins Using De Novo Fragment-Based Protein Structure Predictions
Source: Sci Rep. 2017 Aug 1;7:6999. doi: 10.1038/s41598-017-07156-1 (PMC5539115; doi:10.1038/s41598-017-07156-1)

Predictions of Backbone Dynamics in Intrinsically Disordered Proteins Using De Novo Fragment-Based Protein Structure Predictions

Tomasz Kosciolek, Daniel Buchan and David T. Jones*

Bioinformatics Group, Department of Computer Science, University College London, Gower Street, London WC1E 6BT, United Kingdom

*e-mail: d.jones@cs.ucl.ac.uk

Supplementary Text S1. Examples of different quality FRAGFOLD-IDP predictions.

To get a better intuition about the quality of FRAGFOLD-IDP predictions and the meaning behind R_S values, here we discuss in more detail examples of poor, medium and excellent FRAGFOLD-IDP predictions. Disorder profiles presented in the main manuscript Figure 1 are reproduced here for convenience and clarity.

An example of a poor prediction is 1SIY – lipid transfer protein 1 (Figure 1). The prediction achieves an R_S value of 0.21. Indeed, the disorder profile is not informative. Although the disordered region between residues 50 and 62 is correctly identified, the noise coming from false positives makes it lost in 4 other highly disordered regions predicted by FRAGFOLD-IDP. Also, the short disordered region around residue 20 is completely missed in the prediction.

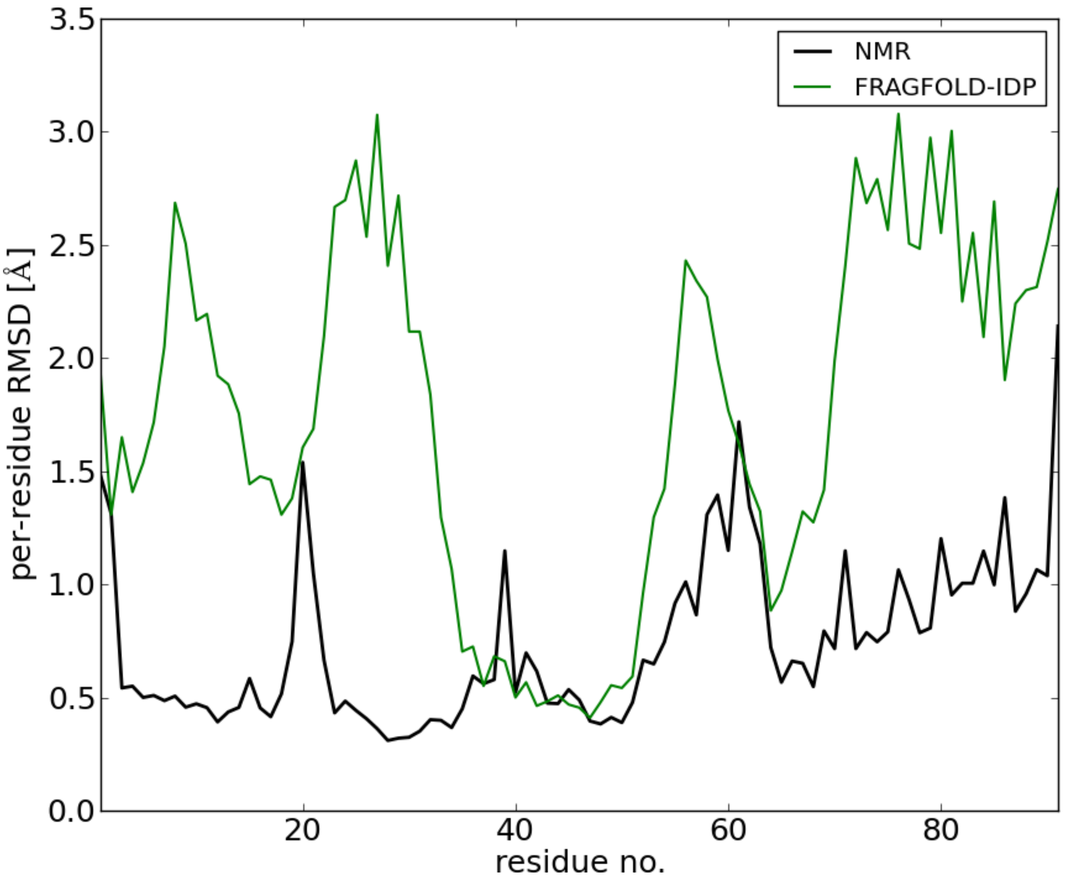

Figure 1. Disorder profile of a poor FRAGFOLD-IDP prediction (1SIY; R_S = 0.21).

An example of a medium quality prediction is 1P94 – ParG protein (Figure 2). The prediction achieved R_S = 0.54, which is close to the median value of the predictions on the entire dataset. Here, FRAGFOLD-IDP correctly identifies first 15 residues as highly disordered, but underestimates the breadth of this region, which spans 35 residues. Finally, the predictions from around residue 48 to 76 are correctly identified as ordered and the disorder profile shows low per-residue RMSD values.

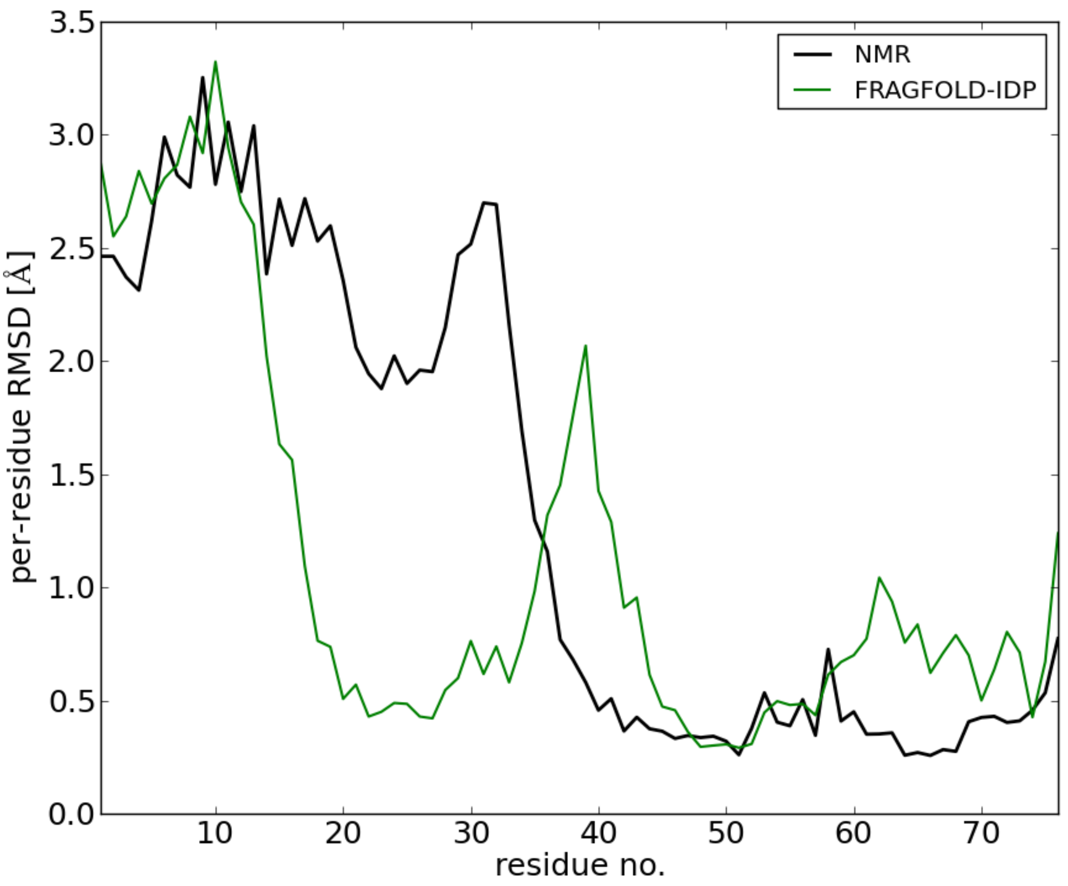

Figure 2. Disorder profile of a medium quality FRAGFOLD-IDP prediction (1P94; R_S = 0.54).

An example of an excellent prediction is 2KJV – ribosomal protein S6 (Figure 3). It achieves an R_S value of 0.82. FRAGFOLD-IDP captures all of the features of the NMR disorder profile remarkably well. The large disordered region between residues 40 and 60 is well reproduced, although FRAGFOLD-IDP slightly overestimates it, extending the region to around residue 35. The C-terminal region (residues 82-101) is also slightly overestimated and in FRAGFOLD-IDP it starts around residue 79. Finally, a small medium disorder region around residue 10 is captured by FRAGFOLD-IDP, but it spans from residue 1 to 15, instead of residue 7 to 12. The

increase in per-residue RMSD signal could be partially attributed to the way sliding window (window size = 10) superposition works, i.e. from residues 1 to 9 there are less averaging steps, because of the sliding window size – residue 1 is superposed only once, residue 2 twice, etc.

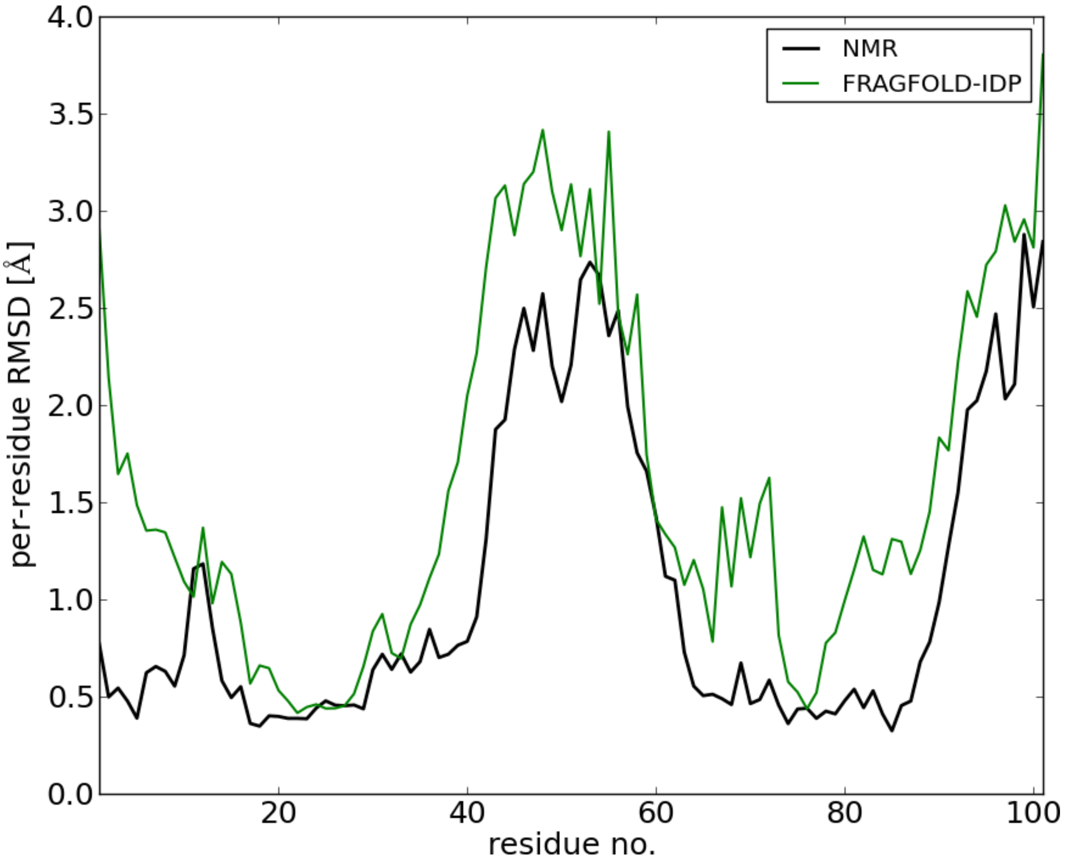

Figure 3. Disorder profile of an excellent FRAGFOLD-IDP prediction (2KJV; R_S = 0.82).

Supplementary Text S2. Outliers.

The results of the outliers are gathered in Table 1. The set contains proteins shorter than an average in the dataset (75 residues in outliers and 105 residues in the dataset), but have a typical disorder content (29% in outliers, 33% in the dataset). FRAGFOLD-IDP R_S is the output of the FRAGFOLD-IDP method, best cluster R_S represents the highest R_S result generated on the same set of models as FRAGFOLD-IDP R_S, but selecting the highest R_S among the clusters generated by PFClust. Top and median R_S values come from 1,000 random ensembles generated from the same raw ensemble, as previously. Naïve R_S are the results of the naïve approach that uses only secondary structure prediction, but does not require any simulations.

Table 1. Outliers in FRAGFOLD-IDP predictions.

protein	length	% disorder	FRAGFOLD-IDP R_S	best cluster R_S	top R_S	median R_S	naïve R_S
1G6M	62	33.87	-0.55	-0.16	-0.23	-0.50	0.26
1K0T	80	36.25	-0.57	-0.33	0.19	-0.48	-0.02
1XN7	78	20.51	-0.49	-0.49	0.69	-0.54	0.66
2K02	79	24.05	-0.51	0.67	0.87	0.71	0.72

Two of the cases among the outliers are clearly related to the ensemble extraction method – 1XN7 and 2K02 (Table 1). The final FRAGFOLD-IDP ensemble results are low, but among FRAGFOLD-generated models (raw ensembles) there are some with excellent R_S values (top R_S). In case of 2K02, the poor result can be attributed to cluster selection criteria, as among the PFClust-generated clusters there is one which achieves a very good results (best cluster R_S = 0.67). 1XN7 is a more general ensemble extraction problem, as the clustering algorithms do not extract a high quality cluster at all – both FRAGFOLD-IDP R_S and best cluster R_S are -0.49. However, FRAGFOLD is able to generate better ensembles for this target, with top R_S reaching a very good R_S value = 0.69. Also, the naïve approach deals well with this target (naïve R_S = 0.66).

The remaining cases – 1G6M and 1K0T – are more challenging (Table 1). Although all results – best cluster, top cluster and median results are better than the selected cluster, the R_S values are still very low (the highest R_S for 1G6M = -0.16 and for 1K0T R_S = 0.19). Comparison with the naïve approach hints that some FRAGFOLD problems are likely, as for 1G6M the naïve

approach generated the best result of all of the attempts (naïve R_S = 0.26), and for 1K0T only the top R_S is higher than the naïve result (top random cluster R_S = 0.19). Still, even the naïve calculations produce results far lower than for the 2 cases discussed previously (1XN7 and 2K02).

1G6M is a snake Cobrotoxin II from *Naja kaouthia* (Monocled cobra). It is a mostly beta sheet protein. From the NMR PDB ensemble of 1G6M it can be inferred there are 4 disulphide bridges that constrain the structure making it more ordered (Figure 4). The bridges are evenly spaced (bridge 1: residues 3 & 24; bridge 2: 17 & 41; bridge 3: 43 & 54; bridge 4: 55 & 60) within the protein structure and constrain the loop regions. There are no disulphide bridges in the beta-hairpin region (residues 25-40). Those bridges are the likely cause of poor predictions of the backbone dynamics achieved by FRAGFOLD-IDP.

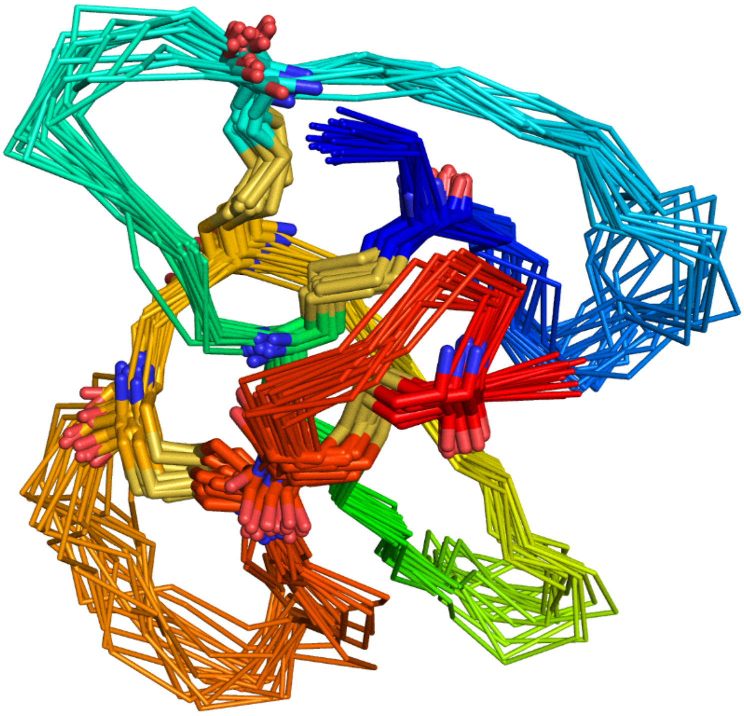

Figure 4. NMR PDB ensemble of 1G6M (Cobrotoxin II). Disulphide bridges are represented as yellow sticks in the ensemble. The rest of the structure is shown in ribbon representation and each conformation in the PDB ensemble is shown as a separate structure.

1K0T is photosystem I subunit PsaC from *Synechococcus sp*. Although the protein passed all of the dataset criteria, it has two inorganic clusters (Fe_4S_4) covalently bound to the protein

(Figure 5). Such modification is likely to alter backbone dynamics of the protein. It can also be confirmed by the fact that other backbone dynamics predictors evaluated fail to significantly improve (e.g. DynaMine R_S = 0.23) over FRAGFOLD-IDP predictions.

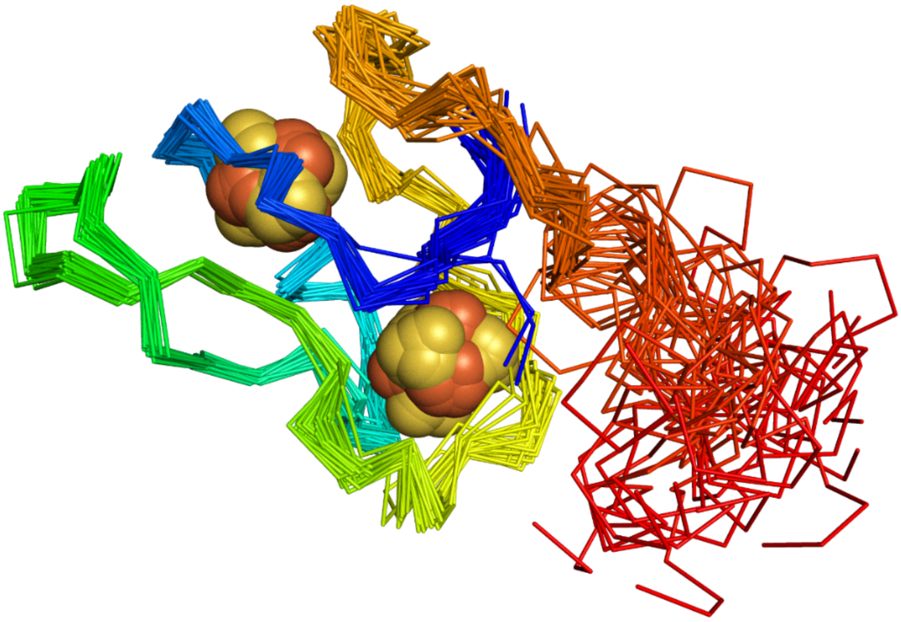

Figure 5. NMR ensemble of 1K0T (photosystem I Subunit PsaC). The ensemble is rainbow-coloured (N-terminus – blue, C-terminus – red). Inorganic (Fe_4S_4) clusters are represented as yellow and orange spheres.

Supplementary Figure S1. Relationship between the disorder content in NMR ensembles and per-CATH class quality of FRAGFOLD-IDP predictions. Top level CATH classification (class) was assigned to each protein. In the case if a given protein was not classified in CATH it was given "none" category. For each CATH class, linear regression fit was also computed. Prediction performance for alpha class (N = 58) is not correlated with disorder content (Pearson's r = -0.04, p = 0.78). Beta class (N = 30) is also not correlated with disorder content (r = -0.02, p = 0.90). Alpha/beta class (N = 60) is negatively correlated with disorder content (r = -0.33, p = 0.01). Few secondary structures class (N = 7) is negatively correlated with disorder content (r = -0.80, p = 0.03), but under-represented, especially in cases with disorder > 40% (2 cases). None class (N = 45) is positively correlated with disorder content, but not statistically significant (r = 0.14, p = 0.37).

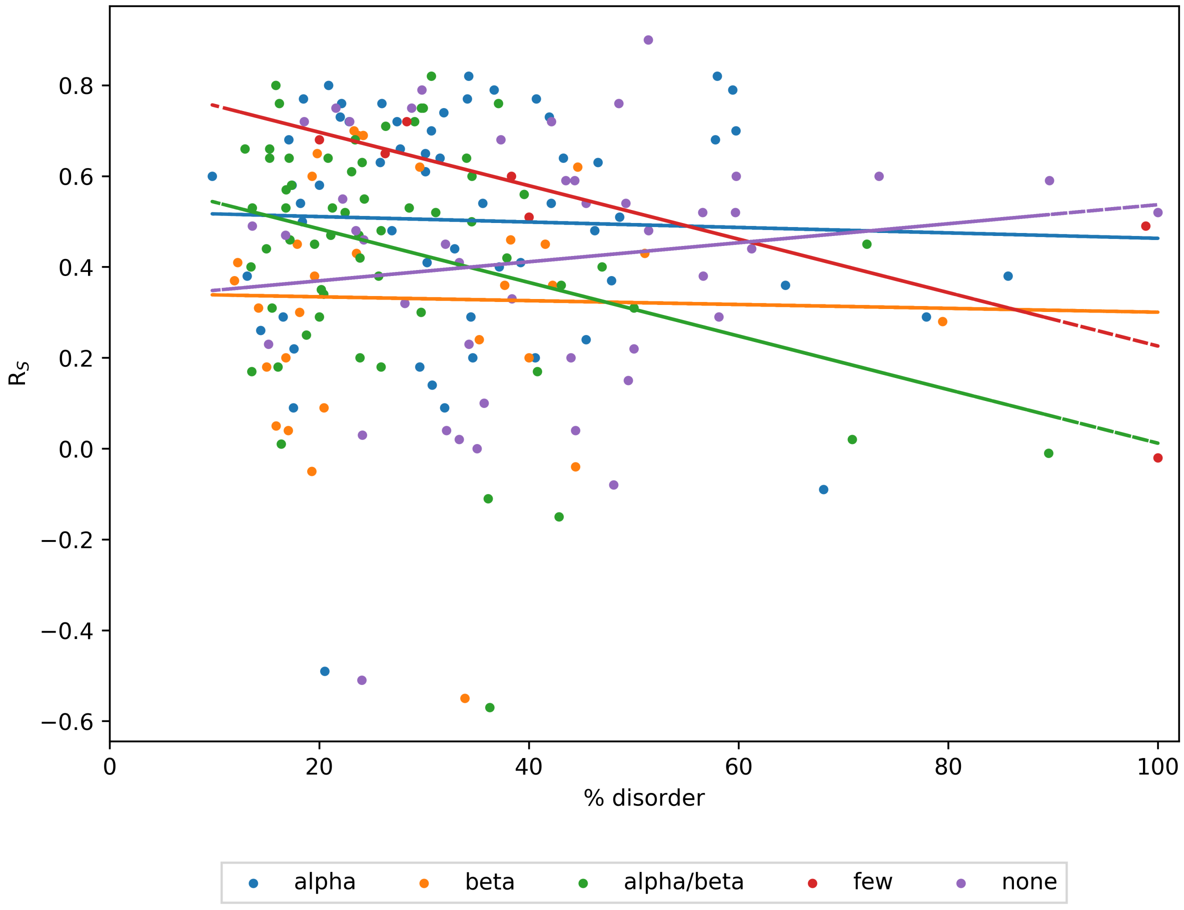

Supplementary Figure S2. Optimisation of the consensus predictor. (A and B) optimisation of the window size using number of features/2 as the number of hidden units. (C and D) optimisation of the window size using geometric mean of the number of input and output units as the number of hidden units. Outliers are shown as red dots.

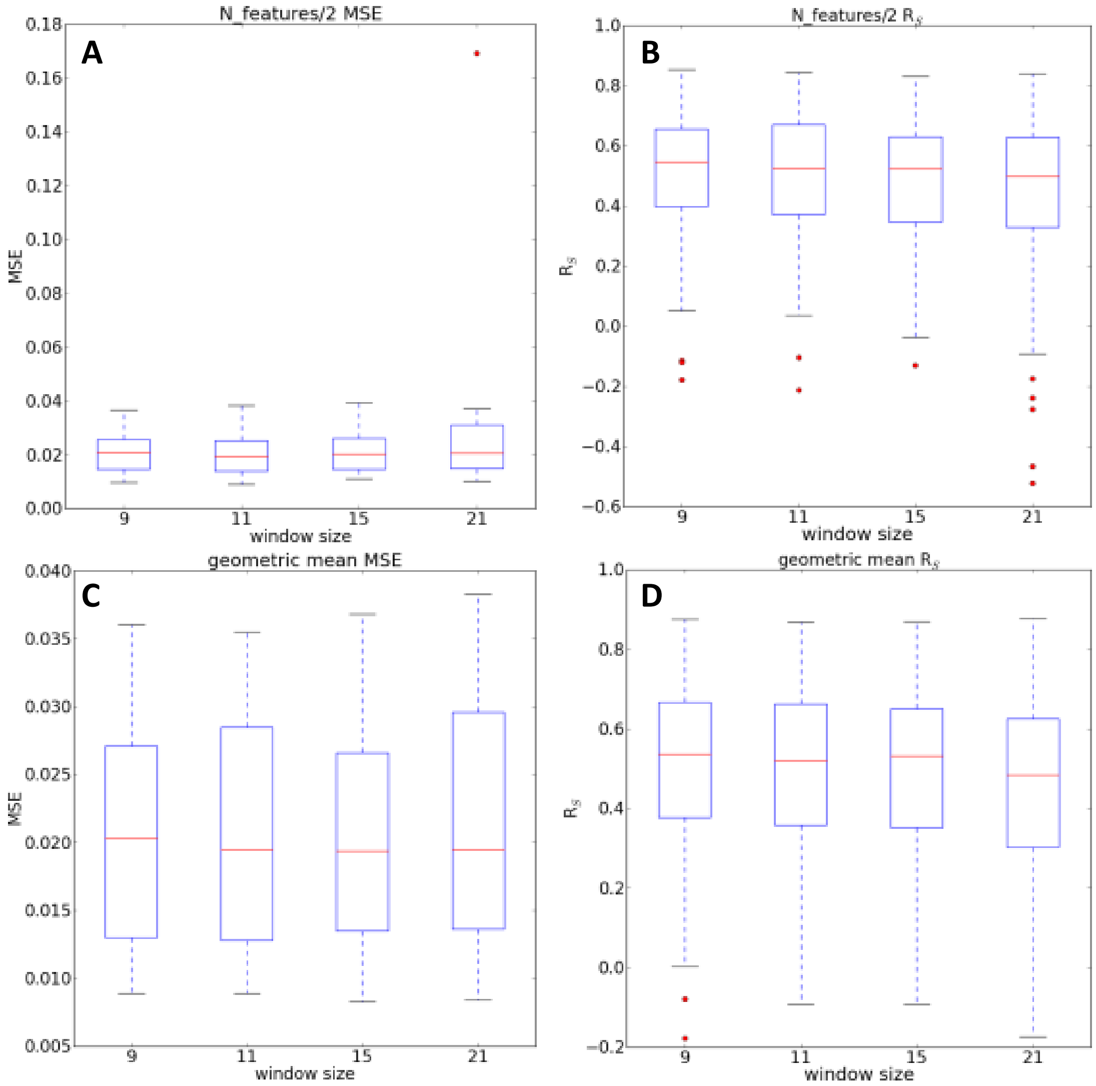

Supplementary Figure S3. Optimisation of the number of hidden units in the consensus predictor.

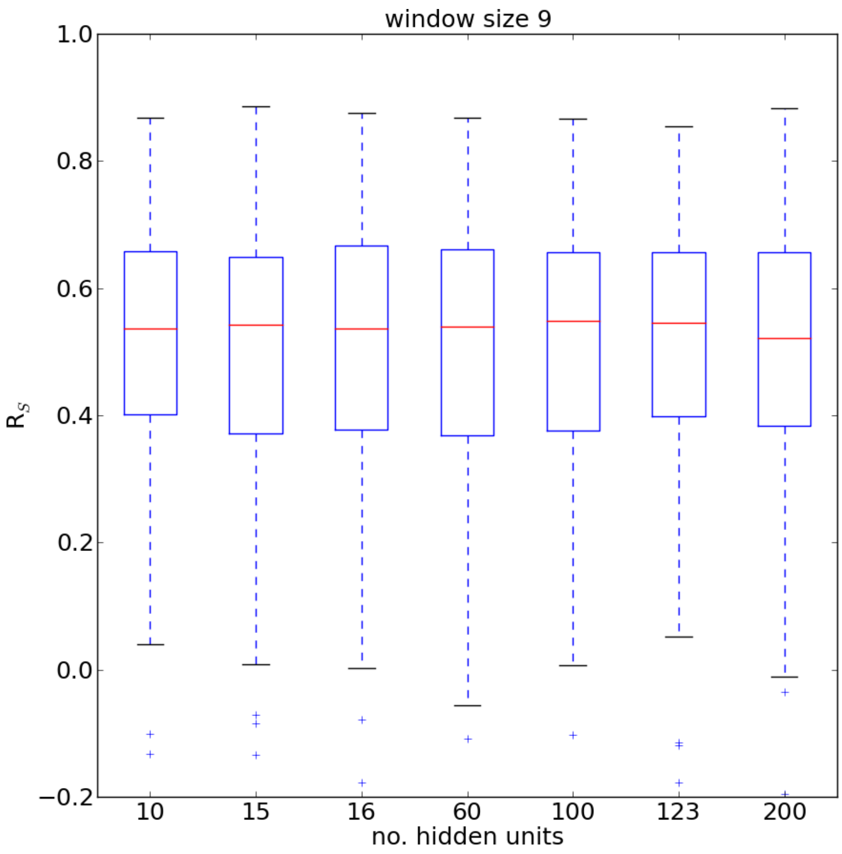

Supplement: Supplementary file 1 — Supplementary Information [file 41598_2017_7156_MOESM1_ESM.pdf]
